# Robot-Assisted Extended Pelvic Lymph Nodes Dissection for Prostate Cancer: Personal Surgical Technique and Outcomes

**DOI:** 10.1590/S1677-5538.IBJU.2015.0055

**Published:** 2015

**Authors:** Porpiglia Francesco, De Luca Stefano, Bertolo Riccardo, Passera Roberto, Mele Fabrizio, Manfredi Matteo, Amparore Daniele, Morra Ivano, Fiori Cristian

**Affiliations:** 1 Divisione di Urologia, Dipartimento di Oncologia, Università di Torino, San Luigi Gonzaga, Regione Gonzole 10, 10043 Orbassano (Torino), Italia; 2 Divisione di Medicina Nucleare, Dipartimento di Internal Medicina, Università di Torino, Ospedale San Giovanni Battista, Corso AM Dogliotti 14, 10126 Torino, Italia

**Keywords:** Complications [Subheading], Laparoscopy, Prostatic Neoplasms, Lymph Nodes, Lymph Node Excision, Surgical Procedures, Operative

## Abstract

**Objective:**

Extended pelvic lymph nodes dissection (EPLND) allows the removal of a higher number of lymph nodes than limited PLND. The aims of this study were to describe our robot-assisted EPLND (RAEPLND) technique with related complications, and to report the number of lymph nodes removed and the rate of lymph nodal metastasis.

**Materials and Methods:**

153 patients underwent RAEPLND prior to robot-assisted radical prostatectomy (RARP). Indications were defined according to Briganti nomogram, to predict risk of lymph-nodal metastasis. Lymphatic packages covering the distal tract of the common iliac artery, the medial portion of the external iliac artery, the external iliac vein and the internal iliac vessels, together with the obturator and the presacral lymphatic packages were removed on both sides.

**Results:**

Median preoperative PSA was 7.5 ng/mL (IQR 5.5–11.5). Median operative time was 150 min (135–170). Median RAEPLND alone operative time was 38 min (32.75–41.25); for right and left side, 18 (15–29) and 20 min (15.75–30) (p=0.567). Median number of lymph nodes retrieved per patient was 25 (19.25–30); 13 (11–16) and 11 (8–15) for right and left side. In 19 patients (12.41%) metastasis was found at the level of pelvic lymph nodes. Median number of positive lymph nodes was 1 (1–4.6) per patient. Complications occurred in 11 patients (7.3%).

**Conclusions:**

the number of lymph nodes removed was comparable to published data about open series, allowing the increase of detection rate of lymph nodal metastasis for minimally invasive approach without compromising complications' rate if performing the procedure following reported technique.

## INTRODUCTION

Pelvic lymph nodes dissection (PLND) is considered the surgical standard for staging of prostate cancer (PCa). The nomenclature and anatomic boundaries of PLND vary. Limited PLND is defined by many surgeons as the removal of lymphatic packages along the external iliac artery and vein, obturator fossa, and obturator nerve (1, 2). In case of extended PLND (EPLND), additionally, lymph nodes along the internal iliac artery together with presacral lymph nodes (the so called “superextended” PLND) are removed too (2–12). Indications of PLND vary: The European Association of Urology (EAU) guidelines recommend performing PLND in all men with a risk of lymph node invasion >5% based on the updated Briganti nomogram (13).

The American Urological Association (AUA) guidelines consider that PLND should be reserved for patients with higher risk of nodal involvement with no clear cut-off (14).

For these reasons some controversies have risen about the appropriateness of limited PLND as a staging tool and more recently increasing evidences support EPLND if PSA level is >10ng/mL or the Gleason Score is ≥7 (3, 5, 7, 10).

It is known that patients affected by high-risk PCa have a risk of lymph nodal metastasis of about 38% (10) but available literature data are derived from open and pure laparoscopic experiences.

Since the introduction of robotic Da-Vinci® system in urologic surgery, robot-assisted radical prostatectomy (RARP) has been becoming an increasingly popular procedure throughout Europe and the United States (10, 15–19).

In parallel with the beginning of RARP case-studies, experiences with robot-assisted PLND have started. The intraoperative magnification together with the higher degrees of freedom in movements allowed by robotic system have boosted performing of EPLND from the beginning in the majority of centers specialized in radical prostatectomy. To date, literature still lacks data about robot-assisted EPLND.

In our study, the primary aim was to describe our surgical technique for RAEPLND and to report surgery-related complications with discussion about how to prevent them; the secondary aim was to report the number of lymph nodes removed by this technique and the detection rate and location of lymph nodal metastasis.

## MATERIALS AND METHODS

From January 2011 to December 2013, 153 patients consecutively underwent RAEPLND for PCa. Indications for RAEPLND were given according to the updated nomogram for prediction of lymph nodes invasion (LNI) by Briganti et al. (13). Patient's demographics were collected and reported in Table-1. All patients preoperatively underwent staging examinations by computed tomography (CT) scanning and/or magnetic resonance imaging (MRI) of the abdomen and pelvis. In patients with a preoperative serum PSA level above 20ng/mL, a total-body bone scanning was performed. Routine postoperative imaging assessment included ultrasonography at 1 month and three months to evaluate surgery-related complications.

**Table 1:** Patient characteristics.

No. of patients		153
Median age, yr (IQR)		64 (59–68)
BMI, median (IQR)		26 (24–28.1)
Preoperative PSA, median, ng/mL (IQR)		7.5 (5.5–11.5)
**Clinical T stage, No (%)**		
	T1	75 (49.0%)
	T2a	31 (20.3%)
	T2b	32 (20.9%)
	T2c	9 (5.9%)
	T3	6 (3.9%)
**Preoperative Gleason Score, No (%)**		
5		2 (1.3%)
6		22 (14.4%)
7a (3+4)		40 (26.2%)
7b (4+3)		39 (25.5%)
8		41 (26.7%)
9		9 (5.9%)
10		0

### 

#### Technique step-by-step

**Figure 1:**
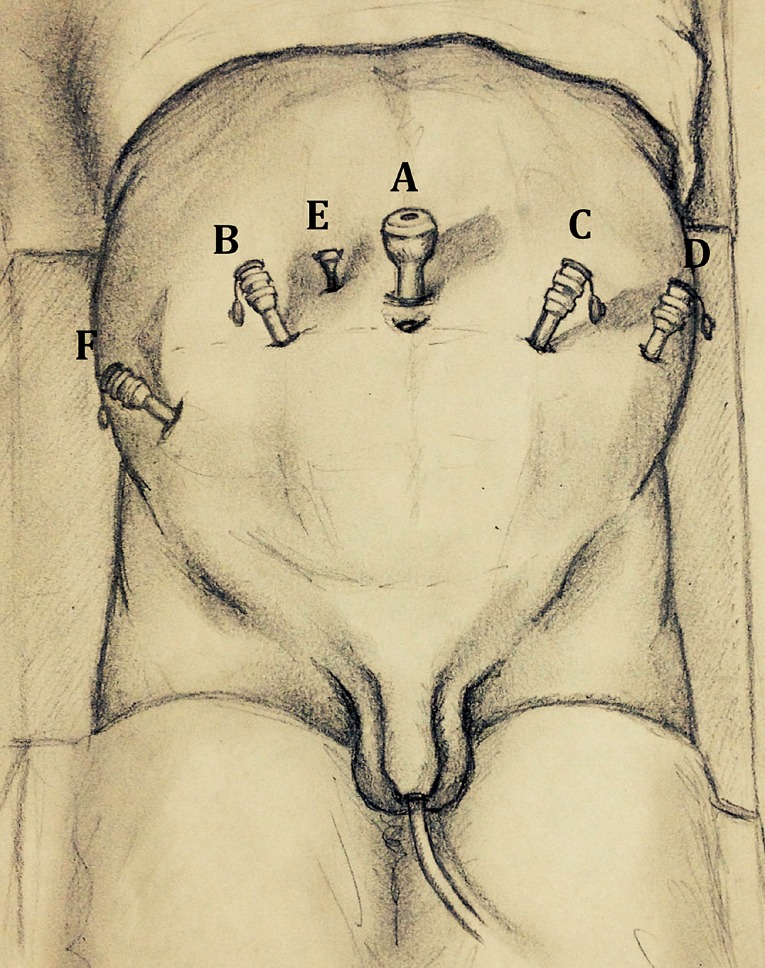
A 10 mm trocar for the camera is placed 20mm above the umbilicus (A). Three 8 mm robotic trocars are placed pararectal on the right and on the left side (B, C), another 8 mm trocar is placed 80mm laterally on the left side (D). Two assistant trocars are placed: one 5 mm trocar between the camera and right working trocar (E) and one 12 mm trocar medial and cranial to the right anterior superior crest (f).

All procedures were performed by transperitoneal approach. Patient was placed in a 30º Trendelenburg position. Using a four-arm Si HD Da Vinci robotic system (Intuitive Surgical, Sunnyvale, CA, USA), six trocars were placed. A 10-mm port for the camera was placed just cranially the umbilicus through a midline incision; two 8 mm ports for the robotic working instruments were placed on the right and left pararectal lines at their intersection with umbilical line. The third 8 mm port (for the fourth robotic arm) was placed 8 cm laterally to the left robotic port. Two additional trocars were placed for the assistant: the first one (5-mm) placed between the camera and the right robotic port; the second one (10 mm) about 8 cm laterally to the right robotic trocar and about 4 cm cranially to the right anterior superior iliac spine (see Figure-1).

The anatomic landmarks of our technique of RAEPLND were the umbilical artery and the iliac vessels.

The anatomical limits were the bifurcation of the common iliac arteries, including the identification of the ureter cranially, the Cloquet's lymph node caudally, the external iliac artery laterally, and the bladder wall medially. The lymph nodes dissection included the lymphatic packages located at the angle between external and internal iliac artery and along the obturator nerve. The dissection was performed by bipolar forceps and monopolar scissors. We here report the specific steps of the procedure:

1-Conventionally, the RAEPLND is begun on the right side. After mobilizing the sigmoid colon, PLND starts with the incision of peritoneum, laterally to the umbilical ligament overlying the common iliac artery and parallel to the external iliac artery until the ureter.

Incision is performed at the level of the pubic bone until the crossing of the ureter with common iliac vessels. External iliac vessels are identified and exposed. Vas deferens is identified and sectioned.

A blunt dissection is performed (preserving pre-vesical fascia) in order to enlarge the operative field among lateral bladder wall, iliac vessels and lateral pelvic wall. The peritoneum covering hypogastric vessels and sacrum is medialized.

2-The ureter is identified at its crossing with common iliac artery, dissected, suspended (if necessary by using a vessel loop) and then displaced (we underline that vessel loop is generally used on the left side only because on the right side ureter adheres to peritoneum so that medialization of peritoneum itself is enough for ureter displacement). The operative field is now well prepared. We underline that presacral area is easy to get and dissect on the right side, while on the left side sacrum promontorium is usually covered by common iliac vein that limit the intraoperative vision and the dissection of presacral lymph nodes.

**Figure 2:**
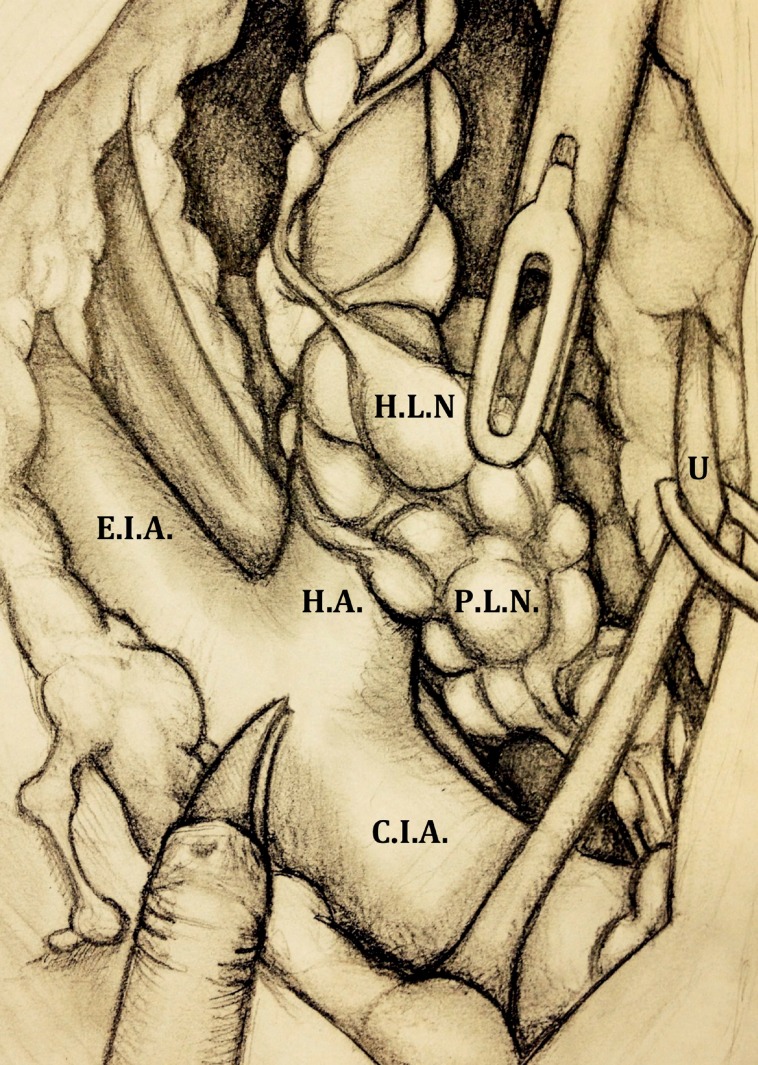
Overview after suspension of the ureter (U) and removal of the fibrofatty tissue overlying the distal portion of the common (C.I.A) and external (E.I.A) iliac vessels (the bifurcation of the common iliac artery is now visible); the presacral (P.L.N) and hypogastric (H.L.N) lymph nodes are identified and dissected (left side). H.A hypogastric artery.

Once splitted the fibro-fatty tissue overlying the distal portion of the common and external iliac vessels, common iliac artery and its bifurcation are visible. The fibro-fatty tissue containing the lymphatics overlying the internal iliac artery, its medial vesical branches and the presacral lymph nodes are identified and dissected (Figure-2).

**Figure 3:**
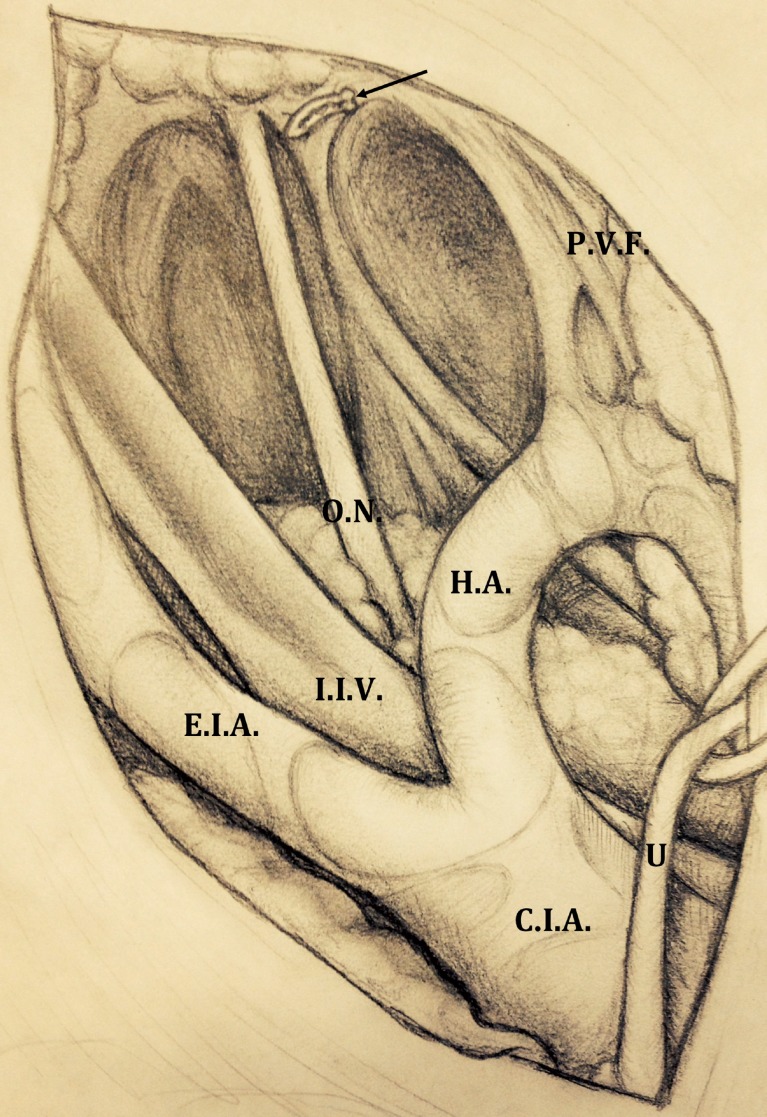
The dissection is carried out from the pubic bone (a Hem o-lok clip is placed just cranially to the Cloquet lymph node, see the arrow) to the crossing of the ureter over the common iliac artery. Then, the obturator fossa is reached and the lymph nodes are progressively dissected until complete exposition of obturator nerve (O.N) is achieved. prevesical fascia (P.V.F). Internal iliac vein (I.I.V).

3-The external iliac lymph nodes are progressively dissected. The dissection of the external iliac packages starts with the division of the adventitia overlying the external iliac vein distally. Then dissection is carried out from the crossing of the ureter over the common iliac artery until the pubic bone at the level of circumflex vein that usually is preserved and dissected (one Hem-o-lok clip is placed just cranially to the Cloquet's lymph node, preserved in order to prevent lymphocele and lymphoedema) (Figure-3). Lateral limit of such a dissection is the medial portion of the external iliac artery: the tissue covering the lateral part of the artery is spared in order to prevent lymphoedema.

4-Then the obturator fossa is reached and the lymph nodes are progressively dissected until complete exposure of obturator nerve. Dissection is here performed with care in order to avoid any neural injury.

The dissection is started at the angle between the external iliac vein and the pubic bone. The lymphatic package is dissected beneath the external iliac vein, proceeding until the pelvic side wall, which is the lateral limit of the dissection. The proximal attachments of the lymphatic packages are dissected by using a combination of either sharp or blunt dissection, paying attention in order to avoid any sharp, blunt, or thermal injury to the obturator nerve. The same technique is performed contra-laterally.

**Figure 4:**
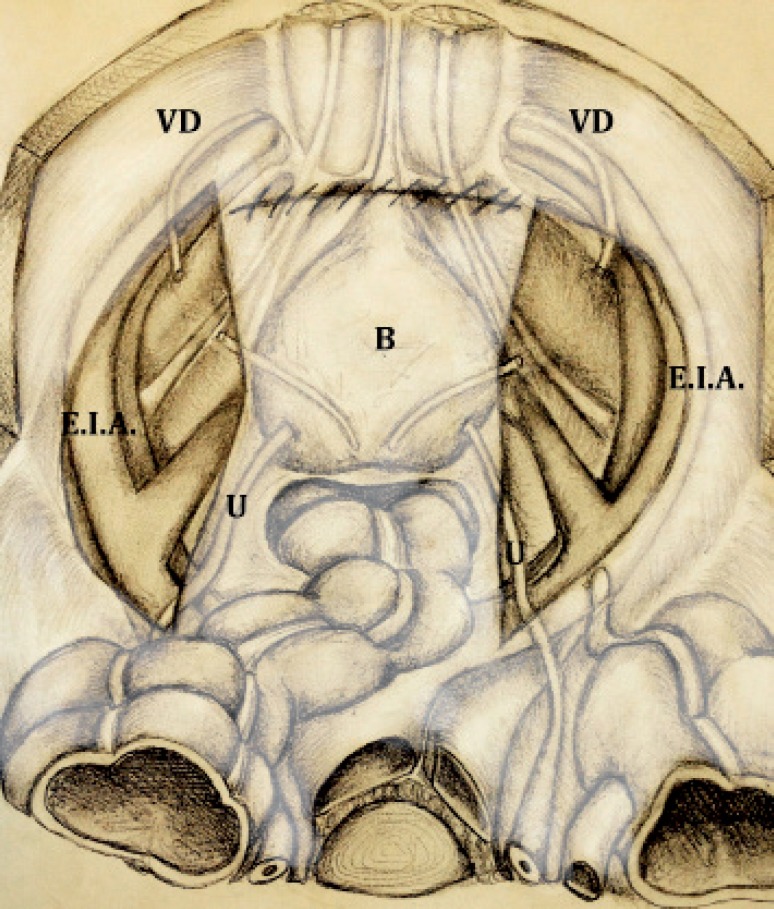
At the end of the peritoneum suture the peritoneal cavity and retropubic space do not communicate thanks to prevesical fascia. Bladder (B). Vas deferens (V.D).

5-Once prostatectomy and its reconstructive phase is completed, the anterior peritoneum is sutured by using a running 3/0 “barbed” suture. At the end of the suture the peritoneal cavity and retropubic space are not in communication yet thanks to previously preserved pre-vesical fascia (Figure-4). Bilateral incisions of peritoneum done in order to perform RAEPLND are not sutured: at the end of the procedure, reconstruction is performed only at midline where parietal peritoneum covers the retropubic space.

Two drains are placed: one intraperitoneally and one, extraperitoneally, in the Retzius space: they are usually removed at the 1^st^ and the 2^nd^ postoperative day, respectively.

Specifically for the purpose of the study, in order to assess the location of lymph-nodal metastasis, lymph nodes were sent in two separated packages removed by two laparoscopic endo-catches from both sides (for convention, the right sided ones are secured by hem-o-lok clips to label them): one containing the lymphatics overlying the distal portion of the common, external (only medial portion) and obturator fossa's lymph nodes (they will be disposed on histopathological analysis Table from the caudalest to the cranialest in order to be recognized by pathologist); the other containing the internal iliac artery and the presacral lymphatic packages.

#### Histopathological analysis

A dedicated uro-pathologist performed all histopathological analysis. Tissue was submitted for permanent sectioning. Frozen section analysis was not routinely performed unless that in case of enlarged and/or clinically suspicious nodes.

Pathologic work-up to detect lymph nodes as well as lymph nodal metastases included direct visualization, palpation and standard hematoxylin-eosin staining.

#### Outcome measurements

Skin-to-skin time, RAEPLND (right, left and overall) operative time, estimated blood losses, intraoperative complications (as classified by modified Satava system (20)), postoperative complications (as classified according to the modified Clavien system (21)), duration of hospitalization, catheterization time and transfusion rate were collected and analyzed.

The number and the locations of dissected lymph nodes on each side and the rate of lymph nodal metastases were recorded. The daily amount of drainage secretion (mL) and duration of drainage (days) were registered. In case of patient with a drained volume over 200mL/24 hours, urinary leakage was excluded by creatinine measurement.

### Statistical analysis

The descriptive statistics of patients characteristics are presented as median (IQR, inter quartile range) for continuous covariates, while as frequency (percentage) for categorical ones. No formal inferential test was performed, since the patients came from a single series. The data were analyzed by R 3.0.2 (R Foundation for Statistical Computing, Vienna-A, www.R-project.org).

## RESULTS

Preoperative diagnostics were negative for metastasis in every case. All enrolled patients underwent RAEPLND+RARP. No patient received neo-adjuvant hormonal therapy. Median overall operative time was 150 (IQR 135–170) min. Median RAEPLND alone operative time was 38 (IQR 32.75–41.25) min; for right and left side, 18 (IQR 15–29) and 20 (IQR 15.75–30), respectively, p=0.567. No case was converted to open surgery. Patients were discharged after a median hospital stay of 5 days (IQR 4–7).

Median number of lymph nodes retrieved per patient was 25 (IQR: 19.25–30), specifically 13 (IQR: 11–16) and 11 (IQR: 8–15), right and left side, respectively. In 19 patients (12.41%) metastasis was found at the level of pelvic lymph nodes. Median number of positive lymph nodes was 1 (IQR: 1–4.6) per patient.

In lymph nodal metastatic patients, median PSA level was 8.2 (IQR 5.5–16.5) ng/mL versus 7.3 (IQR 5.3–11.4) ng/mL in negative lymph node-patients. Distribution of metastatic lymph nodes according to pathological stage and final Gleason Score is reported in Tables 2 and 3. Location and number of metastases per anatomic region are reported in Table-4.

**Table 2:** Metastatic lymph nodes according to pathologic staging.

Pathologic T stage		N° of patients	N° of patients with LNI (%)
**Overall**		**153**	**19 (12.4)**
	pT2a	17	0
	pT2b	5	0
	pT2c	41	1 (2.4)
**Total pT2**		**63**	**1 (1.6)**
	pT3a	56	6 (10.7)
	pT3b	34	12 (35.3)
**Total pT3**		**90**	**18 (20.0)**
	pT4	0	0

**Table 3:** Metastatic lymph nodes according to pathologic grading.

Pathologic Gleason Score	N° of patients	N° of patients with LNI (%)
Overall	153	19 (12.41)
5	0	0
6	5	0
7a (3+4)	59	2 (3.38)
7b (4+3)	47	7 (14.89)
8	36	5 (13.88)
9	6	5 (83.33)
10	0	0

**Table 4:** The location and number of metastases per anatomic region.

Anatomic region	Total lymph nodes, No	Metastatic lymph nodes, No (%)	Number of exclusively metastatic lymph nodes in this region
Iliac-obturator left	1611	31 (1.92)	**13**
Hypogastric-presacral right	236	8 (3.38)	**2** * **and 1** **
Iliac-obturator right	1537	22 (1.43)	**7**
Hypogastric-presacral right	524	7 (1.33)	**2** * **and 1** **
Total	**3908**	**68 (1.74)**	-

Evidenced by the pathologist only

* in the hypogastric region or

** in the presacral region

In 11 patients (7.3%), RAEPLND-associated complications occurred: one patient (0.7%) had a temporary and reversible neuropraxia (involving ischiatic and obturator nerve). Ten (6.6%) patients were found to have lymphocele at ultrasonography performed at 1^st^ month postoperatively with a ranging size from 3.2 to 8.0cm. Among them only 5 (3.3%), with a lymphocele with ranging size from 4.5 to 8.0cm, were symptomatic complaining of lower abdominal pain and required percutaneous drainage. Non symptomatic lymphoceles (5 patients) measured lower than 4.5cm maximum diameter and were located in obturator fossa. At 3rd month control these lymphoceles were stable.

The overall median blood loss for RARP including the RAEPLND was 200 (IQR 150–350) mL; 1 patient (0.7%) who had preoperative serum haemoglobin concentration of 9.2g/dL received intraoperative blood transfusions (1 unit) during prostatectomy phase.

## DISCUSSION

In men diagnosed with localized PCa who have opted for surgical treatment by radical prostatectomy, one of the key decision points of urologist is whether to include or not a staging PLND. Whether or not such a procedure has a therapeutic role in prostate cancer management still remains under investigation and even Guidelines do not agree on a uniform approach about it (22).

Up to date, standard imaging technologies (e.g. CT and MRI) are still able to detect enlarged lymph nodes over 1cm in diameter (23).

The rationale for regional lymph nodes dissection in prostate cancer would be the detection of occult micro-metastases for a proper staging of patients and identification of those who might benefit from adjuvant treatments.

Current indications for PLND vary. Increasing evidences support EPLND in patients with PCa if PSA level is over 10ng/mL or Gleason Score ≥7 (2, 9–12). Accordingly, recent data suggest avoiding lymph nodes dissection in low-risk patients (2, 8).

Briganti et al. reported that using a 5% nomogram cut-off for risk of LNI, about 70% of patients would be spared of EPLND, and LNI would be missed in only 1.5% (19). Indeed, we avoided EPLND in all patients with a nomogram-derived LNI risk <5%.

It is known that Briganti et al. predictive nomogram is based on easily available clinical parameters, such as pretreatment PSA, clinical stage, primary and secondary biopsy Gleason score, and percentage of positive cores (13). On the other side, the incidence of lymph nodal metastases is not exclusively dependent by such parameters: quality of surgical performance and extent of PLND have a crucial role. The lack of standardization in terminology and definitions of anatomic dissection landmarks has caused difficult comparisons among published data about this topic.

For this reason, some authors stressed the importance of evaluating the number of removed lymph nodes as a measure of the quality of PLND. The study by Weingärtner et al. on cadavers considered a total number of removed lymph nodes equal to twenty to be sufficient for accurate staging (24). However, given the fact that during the procedure nodes count is not available for surgeon, definition of extended rather than limited PLND is not based on the number of nodes removed but on the anatomical template.

Nowadays EPLND has been widely accepted as the standard of care when a regional surgical staging is required during surgery for PCa (25). It has been described as involving the lymphatic packages along the external iliac artery and vein, the obturator fossa, along the obturator nerve, along the internal iliac artery and presacral lymph nodes. Its metastases-detection performance is two- to three-fold the limited PLND one, increasing the diagnostic value of lymph nodes dissection (11, 15, 19).

Extending PLND template may increase the risk of complications: this have to be counterbalanced by potential benefits. Overall and EPLND-related complications during RARP series are reported in Table-5. As expected the most frequently reported complication in literature was lymphocele formation, proportional with the number of lymph nodes removed (10.3% with >10 nodes versus 4.6% with <10 nodes) (8, 12, 14). Previous reports showed complications' rates for limited and standard PLND ranging from 2 to 9.8%, while EPLND complication's rates vary from 19.8% to 75% (15, 20). Seventy-five percent of complications in EPLND may be due to extensive dissection of lymphatic tissue along the external iliac artery that primarily supplies the lower extremities. Moreover, this area has never been shown to be affected by metastases in previous anatomical studies (7, 8).

**Table:** Overall and EPLND-related complications in RARP series.

Study	Overall, %	Potentially related to EPLND,%	Clavien grade 1–2, %	Clavien grade 3–4, %
Stone et al. (8)	14.6	10.6	Lymphocele, 6 Lymphoedema, 2	1
Feicke et al. (11)	-	7	Lymphatic fistula, 1 Lymphoedema, 2 Lymphocele, 4	0
Patel et al. (17)	12.3	8.2	Lymphocele, 2.1 Lower extremity oedema, 3	0
Zorn et al. (18)	13	5	Lymphocele, 2	Ureteral injury, 1 Bladder injury, 1 Vena cava compression, 1 Pulmonary embolus, 1
Katz et al. (44)	35.1	Limited: 8.1 Extended: 3.1	Neuropraxia, 3 Neuropraxia, 3.1	Deep vein thrombosis, 3 Pulmonary embolus, 1.6
Sagalovich et al. (4)	-	-	Lymphocele, 2.1	0
Davis et al. (4)	-	5	Lymphocele, 4 Neuropraxia, 3 Lower extremity oedema, 4	1
Present series	8.0	7.3	Neuropraxia 0.7 Lymphocele 3.3	Lymphocele 3.3

To reduce or to prevent PLND-associated morbidities, advices regarding meticulous surgical technique have been provided by several authors (8, 9): first, all the lymphatic vessels coming from the lower extremities should be tied by using ligatures instead of clips. In our experience a Hem-o-lok clip only is placed just cranially to the Cloquet's lymph node, preserved in order to prevent lymphocele. Second, all lymphatics lateral to the external iliac artery should be spared in order to prevent lymphoedema. Third, two drainages should be placed, one per side of the pelvis and should not be removed until the total amount of fluid drained is below 50mL per 24 hours.

In the present prospective study we reported our experience with RAEPLND. Transperitoneal approach was chosen in all cases allowing an excellent working space. Our median number of lymph nodes removed was 25, in line with published data on open series (1, 3, 17, 26–30) and comparable to the similar study published by Feicke et al. on robot-assisted approach who reported a median number of lymph nodes removed of 19 (11).

Among the 68 metastatic lymph nodes, 15 (22.05%) of them were localized either in the hypogastric or presacral regions (both not included in the limited PLND). In the vast majority, they were associated to the presence of lymph node metastases in the external/obturator iliac area. In our series, we observed just one patient (5.26%) with positive lymph nodes in bilateral presacral region only and two patients (10.52%) with hypogastric artery positive nodes only.

A dedicated comment about this finding is needed. It could seem this paper is actually an argument for not doing EPLND due to such a large number needed to treat (51:1) in order to record a benefit in undergoing EPLND (just 3 patients out of 153, 1.96%, exclusively found metastasis in hypogastric/presacral region). Moreover we underline that, in 5 patients having metastatic iliac-obturator lymph-nodes, 11 positive hypogastric/presacral region lymph-nodes were retrieved (3.26%). If we sponsor the therapeutic role of lymph-nodes dissection, this is an important finding. On the other side, we daily perform PLND in all patients, with a probability of lymph-nodal metastasis according to Briganti updated nomogram over 5%, which is not so different from this case-study percentages. If we add that our described technique was safe and that EPLND consumed just half an hour in the overall operative time, we believe that this is the right direction. Moreover, by performing hypogastric/presacral lymph-nodes removal, we are convinced about the fact that, in case of biochemical recurrence, further exams such as total body choline-PET will be more reliable.

After more than 100 procedures we suggest a possible solution to the most important surgical matter: the complete reconstruction of peritoneum at the end of RARP, in order to avoid communication between the peritoneal cavity and retropubic space is paramount. We underline that the bilateral peritoneal incisions above the iliac vessels are not deliberately sutured in order to facilitate lymphatic reabsorption by the peritoneum.

We believe that EPLND-related complications occur due to the fact that peritoneal end extraperitoneal space remains in communication at the end of the procedure, and this is particularly true for transperitoneal laparoscopic (pure or robot-assisted) approach.

The strength of our technique is the anatomical reconstruction of the two operative fields, that will be separated again thanks to the peritoneum reconstruction and the previous sparing of prevesical fascia.

The potential advantages are: first, the avoided risk of lymphatic leakage into the retropubic extraperitoneal space; second, the displacement of bowel loops into the retropubic space is avoided; third, eventual future surgeries are facilitated thanks to preserved and separated anatomical spaces. How were we able to remove a high number of lymph nodes in a relatively short operative time? Again thanks to our experience, we here underline some crucial technical steps: first, on the right side, ureter should be identified and then it should always remain inside intraoperative view; in case of any doubts, it should be suspended; second, on the left side, surgeon should know that ureter will not be sufficiently mobilized by medicalization of peritoneum only: indeed it is paramount to suspend it on this side; third, left iliac vessels anatomy is different: hypogastric vein partially covers sacrum: for this reason presacral lymp-nodes dissection is more challenging on this side.

Even if we were not able to perform long-term functional evaluation, our experience taught us that careful presacral lymph nodes dissection (thanks to robotic-system optical magnification) allows better functional outcomes.

The strengths of the paper are the prospective fashion which was designed by; all specimens were analysed by a dedicated expertise uro-pathologist; follow-up for complications occurrence was adequate. On the other side, the study is not devoid of limitations: case-study was a cohort of patients with high–risk prostate cancer selected according to Briganti nomogram for risk of lymph-nodal metastasis; no comparison with alternative technique was performed; the evaluations were confined about technical aspects, complications and how to prevent them. We disclose for unreporting functional outcomes: they will be object of future researches.
